# *Culicoides* Latreille (Diptera: Ceratopogonidae) as potential vectors for *Leishmania martiniquensis* and *Trypanosoma* sp. in northern Thailand

**DOI:** 10.1371/journal.pntd.0010014

**Published:** 2021-12-15

**Authors:** Sakone Sunantaraporn, Arunrat Thepparat, Atchara Phumee, Sriwatapron Sor-Suwan, Rungfar Boonserm, Glenn Bellis, Padet Siriyasatien

**Affiliations:** 1 Program in Medical Science, Faculty of Medicine, Chulalongkorn University, Bangkok, Thailand; 2 Vector Biology and Vector Borne Disease Research Unit, Department of Parasitology, Faculty of Medicine, Chulalongkorn University, Bangkok, Thailand; 3 Department of Agricultural Technology, Ramkhamhaeng University, Bangkok, Thailand; 4 Department of Medical Technology, School of Allied Health Sciences, Walailak University, Nakhon Si Thammarat, Thailand; 5 Research Excellence Center for Innovation and Health Products, Walailak University, Nakhon Si Thammarat, Thailand; 6 Excellent Center for Dengue and Community Public Health (EC for DACH), Walailak University, Nakhon Si Thammarat, Thailand; 7 Research Institute for the Environment and Livelihoods, Charles Darwin University, Darwin, Australia; Centre hospitalier de Cayenne, FRANCE

## Abstract

Biting midges of genus *Culicoides* (Diptera: Ceratopogonidae) are the vectors of several pathogenic arboviruses and parasites of humans and animals. Several reports have suggested that biting midges might be a potential vector of *Leishmania* parasites. In this study, we screened for *Leishmania* and *Trypanosoma* DNA in biting midges collected from near the home of a leishmaniasis patient in Lamphun province, northern Thailand by using UV-CDC light traps. The identification of biting midge species was based on morphological characters and confirmed using the Cytochrome C oxidase subunit I (*COI*) gene. The detection of *Leishmania* and *Trypanosoma* DNA was performed by amplifying the internal transcribed spacer 1 (*ITS1*) and small subunit ribosomal RNA (*SSU rRNA*) genes, respectively. All the amplified PCR amplicons were cloned and sequenced. The collected 223 biting midges belonged to seven species (*Culicoides mahasarakhamense*, *C*. *guttifer*, *C*. *innoxius*, *C*. *sumatrae*, *C*. *huffi*, *C*. *oxystoma*, and *C*. *palpifer*). The dominant species found in this study was *C*. *mahasarakhamense* (47.53%). *Leishmania martiniquensis* DNA was detected in three samples of 106 specimens of *C*. *mahasarakhamense* tested indicating a field infection rate of 2.83%, which is comparable to reported rates in local phlebotomines. Moreover, we also detected *Trypanosoma* sp. DNA in one sample of *C*. *huffi*. To our knowledge, this is the first molecular detection of *L*. *martiniquensis* in *C*. *mahasarakhamense* as well as the first detection of avian *Trypanosoma* in *C*. *huffi*. Blood meal analysis of engorged specimens of *C*. *mahasarakhamense*, *C*. *guttifer*, and *C*. *huffi* revealed that all specimens had fed on avian, however, further studies of the host ranges of *Culicoides* are needed to gain a better insight of potential vectors of emerging leishmaniasis. Clarification of the vectors of these parasites is also important to provide tools to establish effective disease prevention and control programs in Thailand.

## Author summary

*Culicoides* are known to be vectors of several pathogens, including arboviruses and parasites. However, other investigations have demonstrated that *Culicoides* could be involved in the transmission of *Leishmania* and *Trypanosoma* parasites. Our studies demonstrated the first detection of *L*. *martiniquensis* in *Culicoides mahasarakhamense* and *Trypanosoma* sp., which is closely related to avian Trypanosome in *C*. *huffi* from an endemic area of leishmaniasis in northern Thailand. The finding of this work suggested that *Culicoides* biting midges are suspected to be the potential vector of *L*. *martiniquensis* and *Trypanosoma* parasites in Thailand.

## Introduction

*Culicoides* Latreille are minute hematophagous insects belonging to the family Ceratopogonidae [1]. Several species play an important role in the transmission of arboviruses in domestic and wild ruminants, such as Bluetongue virus (BTV), Schmallenberg virus (SBV), African horse sickness virus (AHSV), Epizootic hemorrhagic disease virus (EHDV), and also in humans such as Oropouche virus (OROV) [2,3]. Apart from viruses, biting midges have been implicated as vectors of several protozoa such as *Parahaemoproteus* sp., *Leucocytozoon* sp., *Hepatocytis*, and filarial worms (*Onchocerca cervicalis*, *Dipetalonema reconditum*, *Mansonella ozzardi*, and *M*. *perstans*) [1,4]. Recent studies have demonstrated the capability of *Leishmania* and *Trypanosoma* parasites to develop in biting midges under laboratory conditions [5–9], suggesting they may also play a role in the transmission of these parasites in the field.

Leishmaniasis is a vector-borne disease that is caused by flagellated protozoa of the genus *Leishmania*. The disease is classified as a neglected tropical disease (NTDs) and is endemic in many parts of the world, especially in tropical and sub-tropical regions [10]. Twenty-one species have been described as affecting humans and these mostly belong to the subgenera *Leishmania* Ross 1903, *Viannia* Lainson & Shaw 1987 [11,12], and *Mundinia* Shaw, Camargo & Teixeira 2016 [13]. In Thailand, leishmaniasis is an emerging disease with the first autochthonous visceral leishmaniasis (VL) cases being reported in southern regions in 1996 [14]. The causative agents of autochthonous leishmaniasis in Thailand are *Leishmania martiniquensis* Desbois, Pratlong & Dedet 2014 [15,16] and *L*. *orientalis* Bate & Jariyapan 2018 (previously reported as "*L*. *siamensis*"), which both belong to *L*. subgenus *Mundinia* [17]. The disease has been reported in both immunocompetent and immunocompromised patients, especially in patients infected with human immunodeficiency virus (HIV) and is endemic in the southern, and the northern regions of the country [18].

The traditional vectors of *Leishmania* species are Phlebotomine sand flies (Diptera: Psychodidae), and initial attempts to identify the vectors of leishmaniasis in Thailand have focused on species of sand fly and detected *L*. *martiniquensis* in three species of *Sergentomyia* [18–21], and *Phlebotomus stantoni* [22].

Increasingly, however, ceratopogonid biting midges are being implicated in the transmission of *Leishmania* parasites. Becvar et al. [9] demonstrated that *L*. *enriettii*, *L*. *macropodum*, *L*. sp. strain GH5 from Ghana, *L*. *orientalis*, and four strains of *L*. *martiniquensis* (MAR1, Cu1, Cu2, and Aig1) successfully developed into metacyclic forms in the stomodeal valve (SV) of *C*. *sonorensis*. They additionally demonstrated transmission of *L*. sp. from Ghana, *L*. *orientalis*, and *L*. *martiniquensis* to mice by the bite of infected *C*. *sonorensis*. Dougall et al. [23] observed *Leishmania* metacyclic promastigotes in the midgut of two species of, *Forcipomyia* (*Lasiohelea*) midges in northern Australia. In addition, several reports have found *Leishmania* parasites in wild-collected *Culicoides* spp. [24–26].

Similarly, recent studies have implicated several species of *Culicoides* in the transmission of trypanosomes. Svobodová et al. [6] reported *T*. *bennetii* infection in wild-caught biting midges and demonstrated the development of *T*. *avium* and a species from the *T*. *bennetii* group in *C*. *nubeculosus*. Bernotiene et al. [8] were able to detect an unidentified species of *Trypanosoma* in 4 different species of *Culicoides* and *T*. *avium* in *C*. *segnis*; moreover, they also reported that *T*. *everetti* could develop in *C*. *nubeculosus* and *C*. *impunctatus* under laboratory conditions [8]. These reports suggest that biting midges may play a role in the transmission of these parasites which have traditionally been associated with tabanids, stable fly, tsetse flies, triatomine bugs, and sand flies [27–29].

Despite this mounting evidence, no investigations into the vectors of *Leishmania* and *Trypanosoma* parasites in Thailand have considered the potential role of biting midges. This study aims to assess the vector potential of *Culicoides* for *Leishmania* and *Trypanosoma* parasites in an endemic area in northern Thailand.

## Materials and methods

### Ethics statement

Experimental protocols of this study were approved by the animal research ethics committee of Chulalongkorn University Animal Care and Use Protocol (CU-ACUP), Faculty of Medicine, Chulalongkorn University, Bangkok, Thailand (COA No. 019/2563).

### Biting midge collection and morphological identification

**Fig 1:**
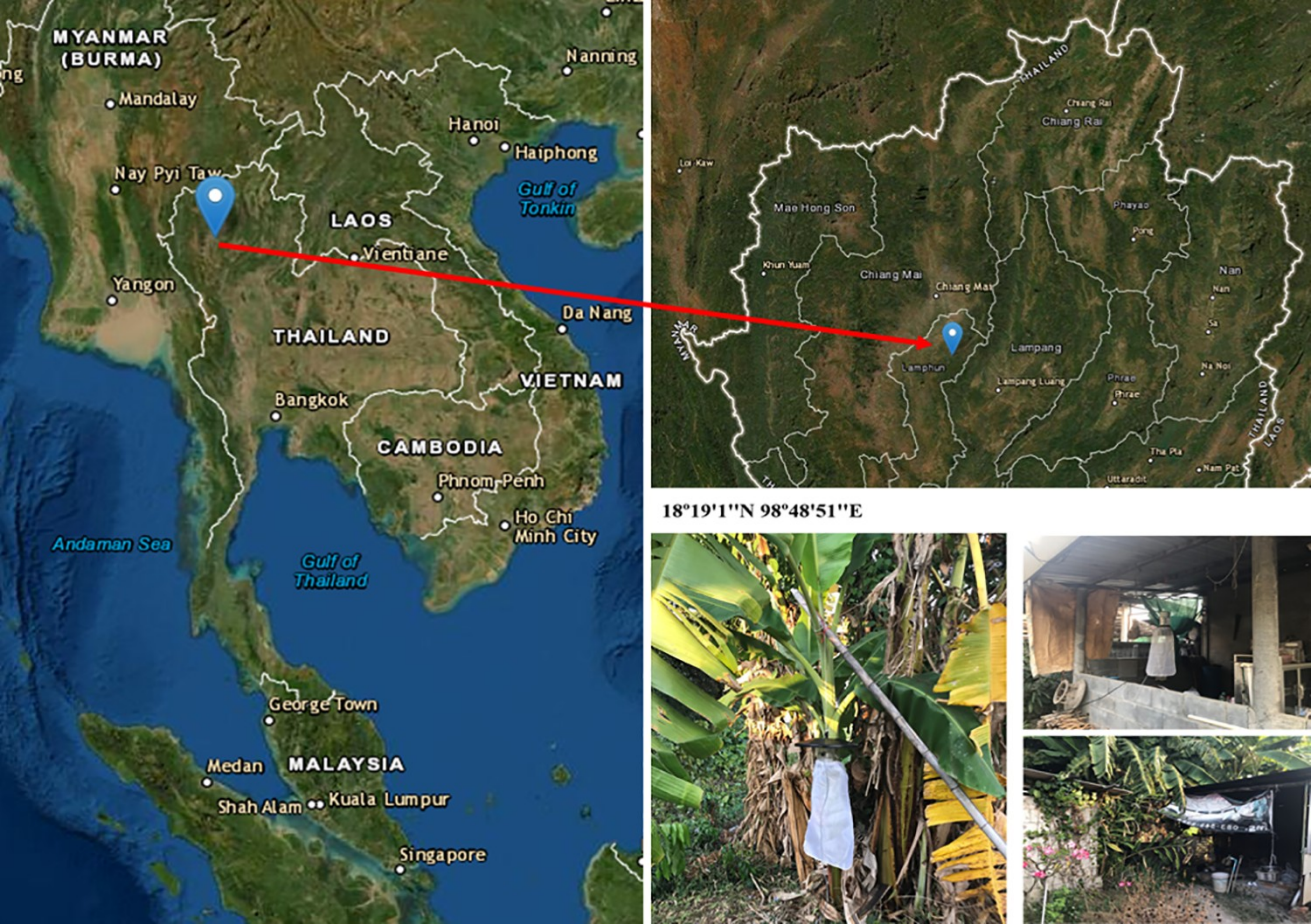
Map of northern Thailand showing the location of biting midge collection sites, including photographs of trap sites in Ban Hong district, Lamphun province, Thailand. (map modified from public domain (https://earthexplorer.usgs.gov/) and photos taken by Padet siriyasatien, corresponding author.

Biting midges were collected near the house of a leishmaniasis patient in Ban Hong district, Lamphun province, northern Thailand (18°19′1″N 98°48′51″E elevation 300 m) (Fig 1). The patient had AIDS associated with the clinical dissemination of cutaneous leishmaniasis. Biting midges were collected live from the house surroundings including chicken coops, stacks of firewood, and banana trees by using Center for Disease Control and Prevention (CDC) miniature light traps (25W bulb) with ultraviolet (UV) light between September 2019 and January 2020. Traps were operated from 6.00 pm to 6.00 am the following morning for two nights per month. All insects were anesthetized at -20°C for 30 min and transported to the laboratory. The female biting midges were examined under a stereomicroscope (Olympus, Tokyo, Japan), and separated from other insects based on morphological appearance. The samples were kept in liquid nitrogen and transported to the Vector Biology and Vector Borne Disease Research Unit, Department of Parasitology, Faculty of Medicine, Chulalongkorn University.

For morphological identification, the head, wings, and genitalia were removed and mounted onto glass slides in a drop of sterilized normal saline and Hoyer’s medium before being examined under a compound microscope [30]. The remaining thorax and abdomen of biting midges were placed in a sterilized 1.5 ml microcentrifuge tube for DNA extraction. Female biting midges were identified using the keys and descriptions of Wirth and Hubert [31].

### DNA extraction

Genomic DNA (gDNA) was extracted from thorax and abdomen of individual midges using a tissue DNA extraction kit (Invisorb Spin Tissue mini kit, STRATEC Molecular GmbH, Berlin, Germany) following the manufacturer’s instructions as modified by Srisuton et al. [21]. The quality of the obtained DNA concentrations of each sample was measured with Nanodrop 2000c (Thermo-Scientific, USA). The DNA sample was stored at -20°C until use in PCR investigation.

### Molecular detection of *Leishmania* and *Trypanosome* parasites

The purified gDNA was used to detect *Leishmania* parasite DNA in conventional PCR with a primer set (LeF 5′-TCCGCCCGAAAGTTCACCGATA-3′ and LeR 5′-CCAAGTCATCCATCGCGACACG-3′) that targets the approximately 379 bp fragment of internal transcript spacer 1 (*ITS1*) region as previously described by Spanakos et al. [32]. PCR reactions were set at a total volume of 25 μl, containing 6 μl of gDNA, 10X PCR buffer, 25 mM of MgCl_2_ (Thermo Fisher Scientific, Walthman, MA, USA), 2.5 mM of dNTPs (GeneAll, Korea), 10 μM of each forward and reverse primer, and 1 unit of *Taq* DNA polymerase (Thermo Fisher Scientific, Walthman, MA, USA). The PCR cycles began with pre-denaturation at 95°C for 5 min, followed by 40 cycles of denaturation at 95°C for 1 min, annealing at 65°C for 1 min, extension at 72°C for 1 min. The final extension was performed at 72°C for 7 min.

In order to detect *Trypanosoma* DNA in biting midges, PCR amplification and reaction methods followed those of Srisuton et al. [21]. In this study, plasmid DNA containing *ITS1* and *SSU rRNA* genes were used as positive controls in *Leishmania* and *Trypanosoma* detection respectively, and sterilized distilled water was used as a negative control.

### Molecular identification of biting midges and blood meal analysis

The biting midge identifications were confirmed by analysis of the partial mitochondrial cytochrome C oxidase subunit I (*COI*) gene. The partial *COI* gene was amplified using the primers LCO1490 and HCO2198 [33] or C1-J-1718 and C1-N-2191 [34]. The PCR reaction and amplification profiles followed the protocols previously described in Harrup et al. [35] and Mathieu et al. [36]. Plasmid DNA containing *COI* gene of *C*. *mahasarakhamense* was used as the positive control and sterilized distilled water was used as a negative control.

The hosts of blood meals in engorged biting midges were identified by two PCR protocols for detection of mammal [37] and avian DNA [38]. Both PCR conditions were described in a previous study by Boonserm et al. [39].

### Sequence analysis and phylogenetic construction

All positive PCR products were cloned into the pGEM-T Easy Vector (Promega, Mandison, WI, USA) followed the method described in Srisuton et al. [21], and the plasmid DNA was then isolated using the Invisorb Spin plasmid mini kit (STRATEC Molecular GmbH, Berlin, Germany) following the manufacturer’s instructions. The purified plasmid DNA was sequenced by the commercial service at Macrogen Inc., South Korea.

All sequences were truncated to obtain consensus sequences of each sample prior to alignment using the ClustalW multiple alignment program in BioEdit Sequence Alignment Editor Version 7.2.5 [40]. The consensus nucleotide sequences were compared to the previously published sequences in the GenBank database using the Basic Local Alignment Search Tool (BLAST) (https://blast.ncbi.nlm.nih.gov/Blast.cgi). Phylogenetic trees were constructed using the maximum likelihood method with 1000 bootstrap replications on Molecular Evolutionary Genetics Analysis software (MEGAX) [41].

## Results

### Biting midge species identification

**Fig 2:**
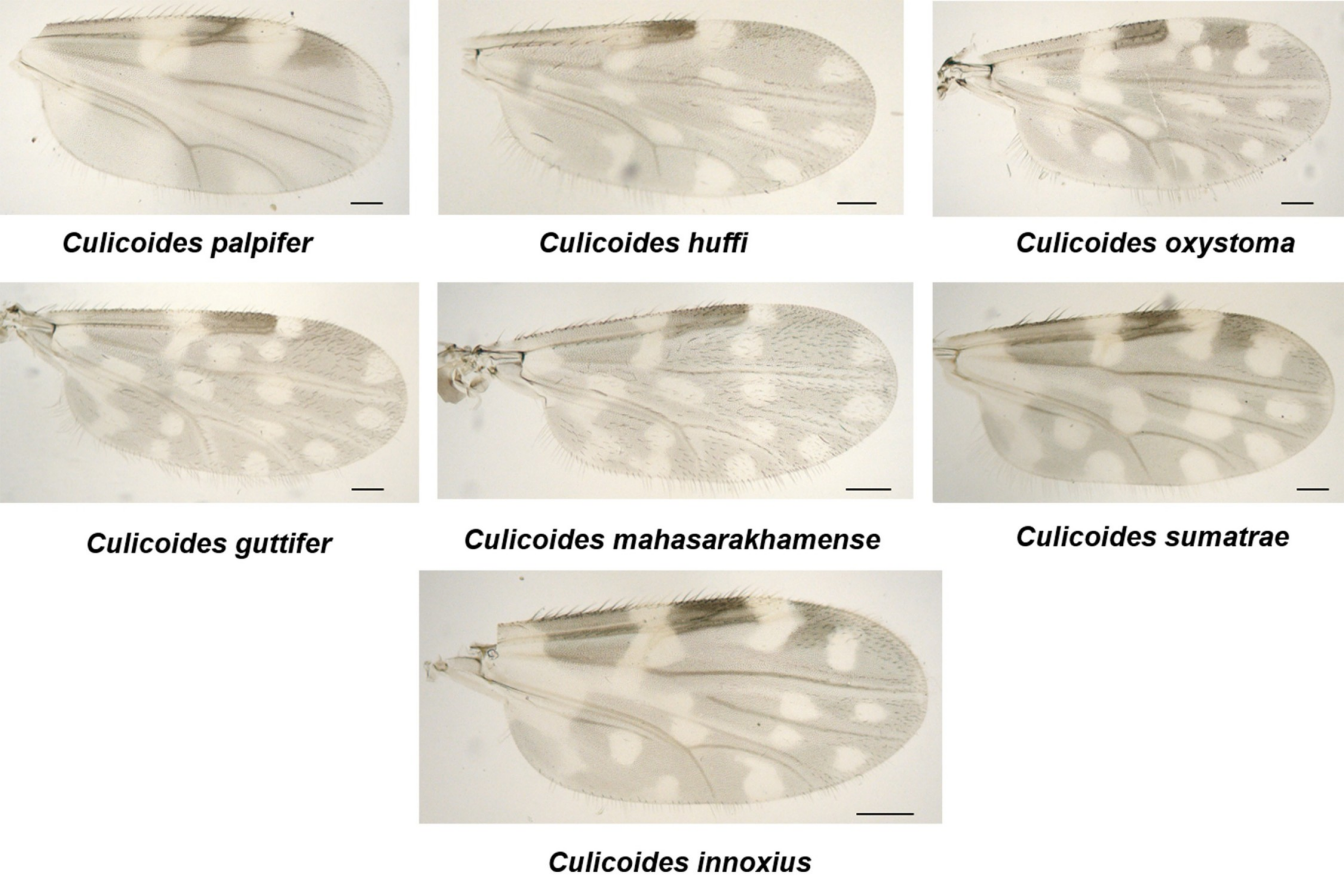
Photographs of representative specimens of the seven species of *Culicoides* found in this study, scale bar: 100 μm.

A total of 223 female biting midges belonging to seven species were captured and identified as *Culicoides mahasarakhamense* (47.53%), *C*. *guttifer* (22.87%), *C*. *innoxius* (12.56%), *C*. *sumatrae* (0.90%), *C*. *huffi* (9.42%), *C*. *oxystoma* (2.24%), and *C*. *palpifer* (4.48%) (Table 1). Fig 2 shows the wing patterns of representative specimens of these species.

**Table 1:** Number of biting midge species collected, tested, and found positive for DNA of *Leishmania martiniquensis* and *Trypanosoma* sp.

Subgenus	Species identification	No. tested	*ITS1*-PCR	*SSU rRNA*-PCR
			*L*. *martiniquensis*	*Trypanosoma* sp.
*Meijerehelea*	*Culicoides mahasarakhamense*	106	3	0
	*Culicoides guttifer*	51	0	0
*Hoffmania*	*Culicoides innoxius*	28	0	0
	*Culicoides sumatrae*	2	0	0
*Clavipalpis* group	*Culicoides huffi*	21	0	1
*Remmia*	*Culicoides oxystoma*	5	0	0
*Trithecoides*	*Culicoides palpifer*	10	0	0
**Total**		**223**	**3**	**1**

**Fig 3:**
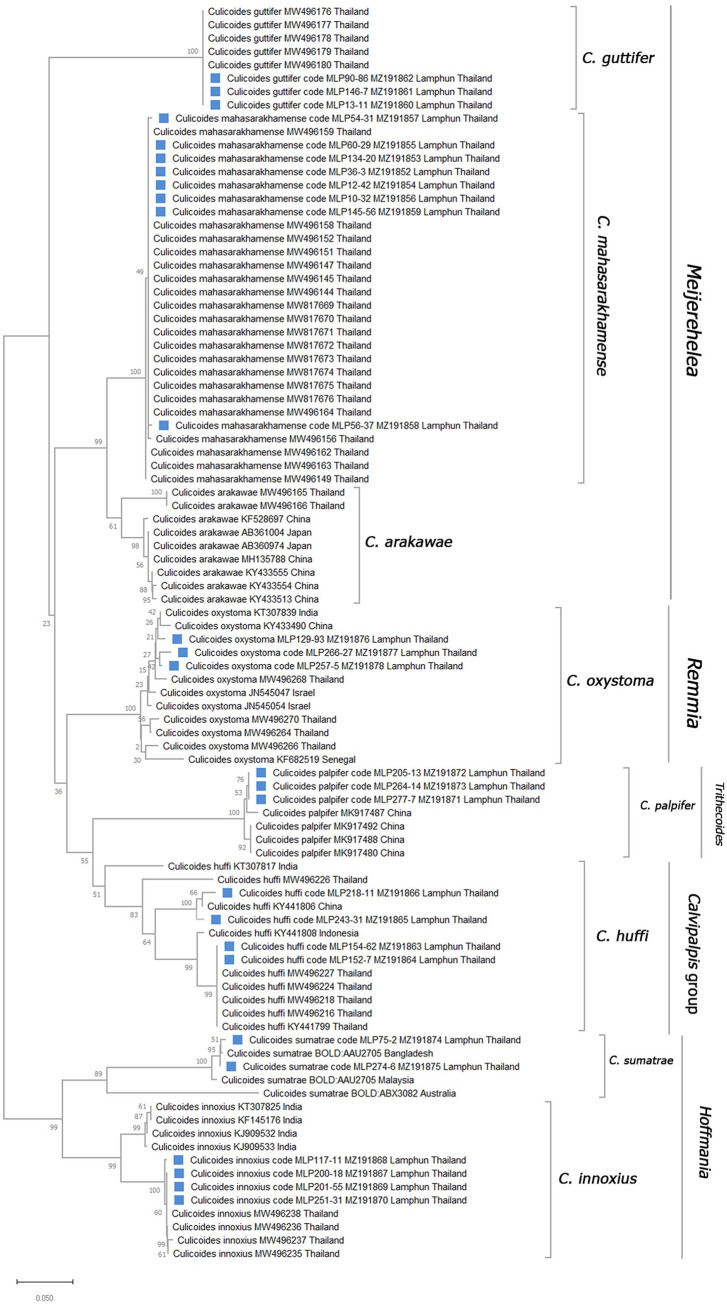
Maximum likelihood (ML) phylogenetic tree using the GTR+G+T model with 1,000 bootstrap replicates of partial *COI* sequences of *Culicoides* biting midges collected in this study and data from conspecific specimens available in GenBank database.

Of the 223 specimens tested for the presence of *Leishmania* and *Trypanosoma* parasites, the DNA of 27 were successfully used for *COI* amplification. These included 23 randomly selected to represent each morphologically identified species and the 4 specimens which were positive for parasite detection. The phylogenetic tree based on *COI* sequences was used to confirm the identifications of all specimens. The phylogenetic tree placed our specimens of *C*. *mahasarakhamense*, *C*. *guttifer*, *C*. *innoxius*, *C*. *huffi*, and *C*. *oxystoma* into clades with conspecific *Culicoides* species previously sequenced from Thailand (Fig 3). These sequences were submitted to the GenBank database under the GenBank accession numbers: MZ191852, MZ191853, MZ191854, MZ191855, MZ191856, MZ191857, MZ191858, and MZ191859 for *C*. *mahasarakhamense*; MZ191860, MZ191861, and MZ191862 for *C*. *guttifer*; MZ191863, MZ191864, MZ191865, and MZ191866 for *C*. *huffi*; MZ191867, MZ191868, MZ191869, and MZ191870 for *C*. *innoxius*; MZ191871, MZ191872, and MZ191873 for *C*. *palpifer*; MZ191874, and MZ191875 for *C*. *sumatrae*; MZ191876, MZ191877, and MZ191878 for *C*. *oxystoma*.

### Detection of *Leishmania*, *Trypanosoma*, and host blood DNA in biting midges

**Fig 4:**
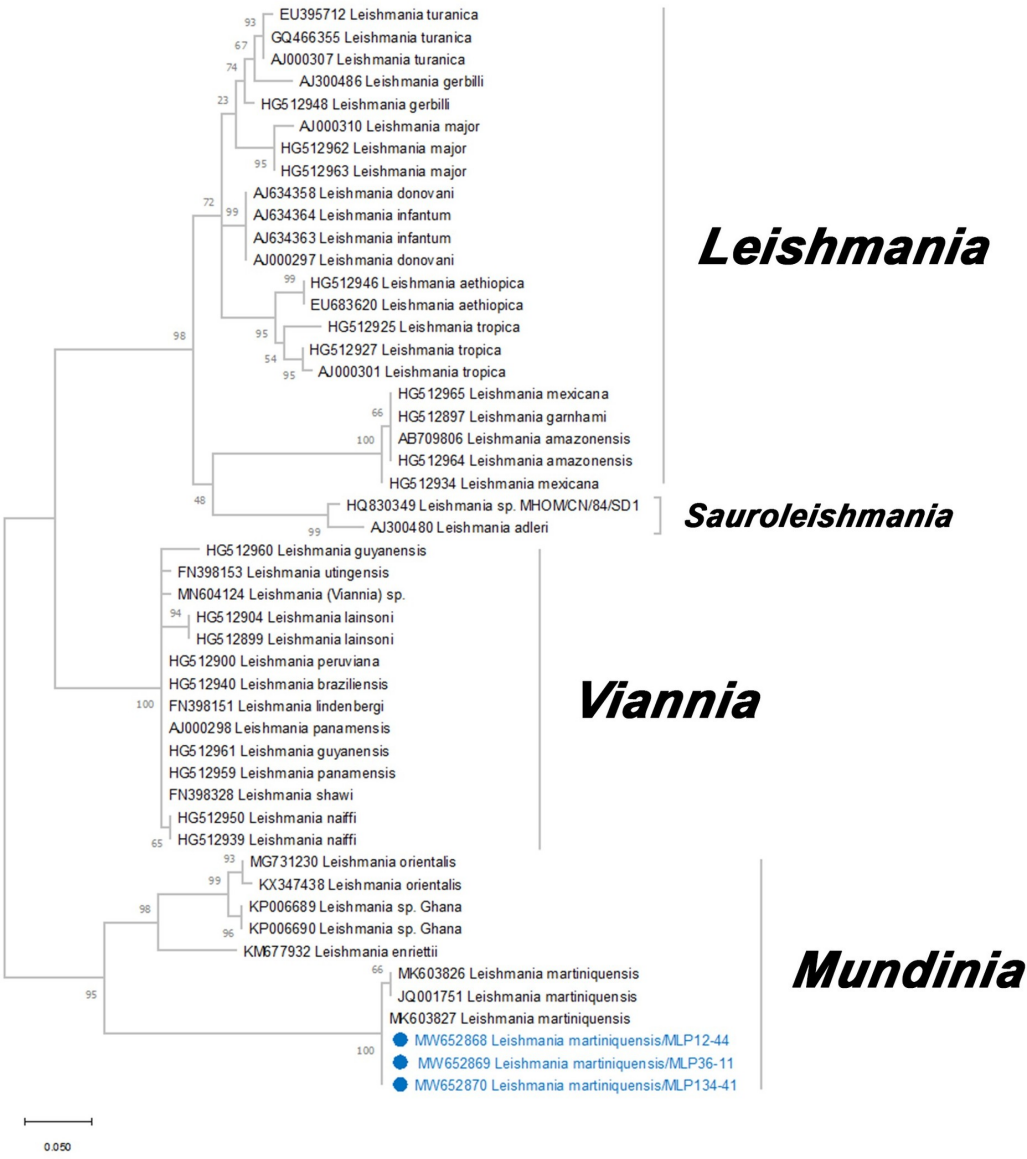
Maximum likelihood (ML) tree based on the K2+I model of nucleotide substitution of *Leishmania* spp. constructed from partial *ITS1* gene sequences. Bootstrap values are based on 1,000 replicates. Blue circles indicated samples that were obtained from this study.

A total of 223 extracted DNA samples from biting midges were tested for the presence of *Leishmania* and *Trypanosoma*. Thirteen of these specimens contained a visible blood meal but no parasite DNA was detected in these specimens. Sequences from the *ITS1*-PCR amplification from 3 specimens of *C*. *mahasarakhamense* (MLP12-44, MLP36-11, and MLP134-41) were consistent with *Leishmania* DNA with nucleotide sequences of *ITS1* gene of 379 bp in length. The BLAST results of these three *ITS1* sequences showed a 100% similarity with *L*. *martiniquensis* sequence data from GenBank accession no. MK603827. This result was supported by the phylogenetic tree which showed that all three *ITS1* sequences were clearly classified under *L*. *martiniquensis* with a bootstrap support value of 100% (Fig 4). The sequences of *L*. *martiniquensis* in this study were submitted to GenBank database (GenBank accession no. MW652868, MW652869, MW652870).

**Fig 5:**
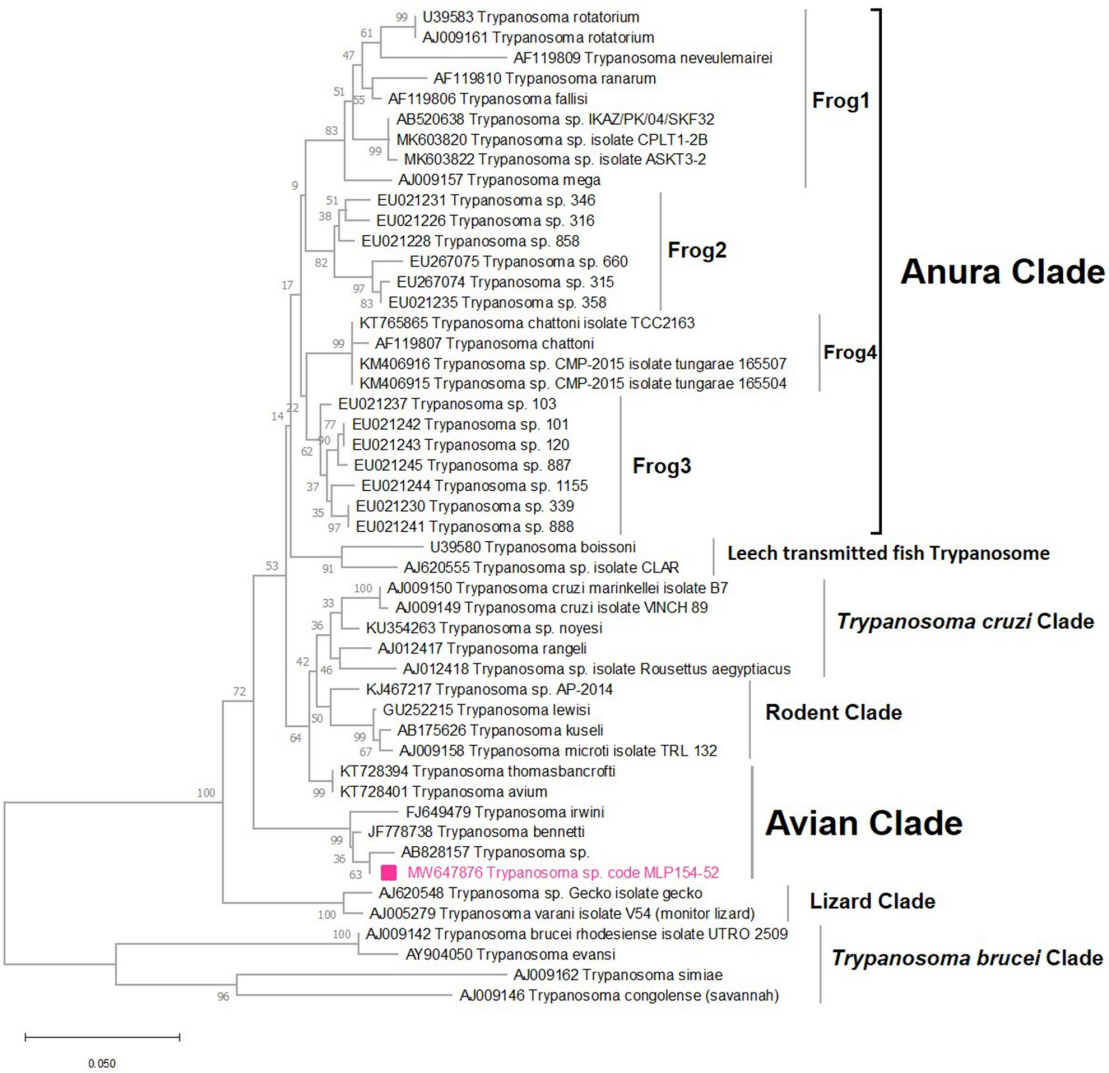
Maximum likelihood (ML) tree created using the TN93+G+I model with 1000 bootstrap replications from partial *SSU* rRNA sequences of *Trypanosoma* parasites from this study and reference sequences available in GenBank. Sequences from this study are indicated with pink squares.

The conventional PCR specific to the *SSU rRNA* gene revealed a band of approximately 934 bp in one sample of *C*. *huffi* (MLP154-52). A BLAST result of this sequence demonstrated a 99.15% match with an unnamed species of *Trypanosoma* (GenBank accession no. AB828157) available in the GenBank database. These sequences were placed within a group of several clades formed by different species of *Trypanosoma* parasites including *T*. *bennetti* (GenBank accession no. JF778738), *T*. *irwini* (GenBank accession no. FJ649479), and the unnamed *Trypanosoma* sp. (GenBank accession no. AB828157) (Fig 5). The Trypanosome sequence obtained from this study was submitted to GenBank database under the GenBank accession number MW647876.

Host blood sources of engorged female specimens of *C*. *mahasarakhamense* (n = 4), *C*. *guttifer* (n = 2), and *C*. *huffi* (n = 7) were tested for the presence of host blood DNA. All of these specimens were found to contain avian DNA with no trace of mammalian DNA detected.

## Discussion

This study is the first to investigate the potential role of biting midges as vectors of *Leishmania* or *Trypanosoma* in Asia. The detection of parasite DNA in field-collected midges satisfies one of the criteria for proving their status as vectors of these respective parasites but the remaining 3 criteria must be proven before a species can be categorized as a proven vector. These 4 criteria cited by Killick-Kendrick [42] are; (1) the vector will feed on man, and if the disease is zoonotic, the animal reservoir host; (2) it will support the development of the parasite after the infecting blood meal has been digested and voided; (3) parasites from wild-caught insects will be indistinguishable from isolates from vertebrate hosts; and (4) the insect will be able to transmit the parasite by bite.

One of these criteria requires an association between the vector, parasite, and reservoir host. For both of the protozoa detected in this study, however, the reservoir host is unclear although *L*. *martiniquensis* DNA was detected in black rats in one study in Thailand [20], and clinical symptoms noted in horses and cattle in Europe [43,44], and horses in North America [45]. There are no reports of leishmaniasis in cattle or horses in Thailand so it may be possible that these are dead-end rather than reservoir hosts. While there are records of the closely related species, *C*. *arakawae*, feeding on large mammals [46] there is no evidence of either *C*. *arakawae* or *C*. *mahasarakhamense* feeding on humans and this would need to be confirmed in *C*. *mahasarakhamense* if this species is to play an important role in the transmission of this parasite to humans.

Similarly, the trypanosome detected in *C*. *huffi* appears to be most closely related to species known to infect birds suggesting this species may also employ birds as reservoir hosts. The known host range of *C*. *huffi* is avian but this has only been reported in a single study [47] although our findings support this report. Additionally, the illustrations of Wirth & Hubert [31] indicate that the structure of the palpus and the number of sensilla coeloconica on the female antennae are consistent with a preference for feeding on birds [48–51].

Blood meal analysis from engorged female *C*. *mahasarakhamense*, *C*. *guttifer*, and *C*. *huffi* revealed that these specimens had all fed on avian hosts. In Thailand, Jomkumsing et al. [47] also found that the blood found in *C*. *mahasarakhamense* (reported as *C*. *arakawae*), *C*. *guttifer*, and *C*. *huffi* belonged to chickens. Presently, there is no evidence of *Culicoides* feeding on humans in Thailand although several species known to attack humans in other countries are present [31]. The potential vector status of *Culicoides* spp. for human diseases; however, indicates that further investigation of the host ranges of biting midges in Thailand is needed, especially in the endemic areas of leishmaniasis in the country.

The detection of *L*. *martiniquensis* in a species of biting midge provides further evidence that species of *L*. subgenus *Mundinia* are associated with this group of insects rather than solely dependant on Phlebotomine sand flies as are other subgenera of *Leishmania* [5,7,23]. *L*. *martiniquensis* DNA has been detected in four species of *Sergentomyia gemmea*, *Se*. *barraudi*, *Se*. *khawi*, and *Ph*. *stantoni* [18–22], suggesting that both midges and sandflies may potentially act as vectors of this parasite. The field infection rate of *L*. *martiniquensis* in *C*. *mahasarakhamense* detected in this study was 2.38% (3 positives from 106 tested) while that for *Se*. *khawi* and *Ph*. *stantoni* were a little higher at 5.41% and 12.5% [21,22], suggesting that *C*. *mahasarakhamense* may in fact play a similarly important role as *Se*. *khawi* and *Ph*. *stantoni* in the transmission of this parasite in Thailand. Field infection rates for the other 2 species of *Sergentomyia* found to harbor *L*. *martiniquensis* are difficult to assess as specimens were tested in pools rather than as individuals [18–20].

Similarly, the detection of a species of *Trypanosoma* in a biting midge supports previous reports that these insects may act as vectors of these parasites [6,8]. The field infection rate of *Trypanosoma* sp. in *C*. *huffi* detected here was 4.76% (1 positive in 21 tested) is which is comparable to rates found in other vectors for example black flies (2.13%) and sand flies (8.58%) but much lower than infection rates for triatomine bugs (55.2%) [21,52–54]. This suggests that *C*. *huffi* is potentially as important a vector as black flies and sand flies.

A total of 100 *Culicoides* species had been recorded in Thailand [55,56]. However, several of these species have been shown to contain cryptic species which are difficult to separate morphologically [48,55,57] so identification requires molecular analysis to clarify the cryptic species involved. Two such species are *C*. *arakawae/ C*. *mahasarakhamense*, and *C*. *huffi* making interpretation of the implications of these species as vectors difficult as it is unclear if published reports on the biology, distribution etc of these species refer to the cryptic species associated with these protozoa or to another of the cryptic species. Currently available information suggests that both *C*. *mahasarakhamense* and the haplotype of *C*. *huffi* found infected with *Trypanosoma* sp. are confined to Thailand. Should the distribution of either of these species be found to be more widely distributed in Asia then the distribution of their respective protozoa may be similarly widespread.

Analysis of *COI* barcodes proved a useful means of confirming identifications of 4 of the 7 species collected in this study. The ML tree analysis however indicates that our sequence data from specimens identified as *C*. *innoxius*, *C*. *huffi*, and *C*. *palpifer* differed to those reported by other workers suggesting of the presence of cryptic species. This is consistent with the findings of other studies in southeast Asia which have identified cryptic species amongst several taxa [47,57]. The *COI* sequence data provided here represent the first Thai data for *C*. *sumatrae* and *C*. *palpifer*.

## Conclusion

This study is the first to report the detection of *L*. *martiniquensis* in *C*. *mahasarakhamense* and *Trypanosoma* sp. in *C*. *huffi* collected from a leishmaniasis endemic area in northern Thailand. Detection of both *L*. *martiniquensis* and *Trypanosoma* parasites in *Culicoides* biting midges suggests that they might be potential vectors of leishmaniasis and trypanosomiasis. Screening of wild-caught *Culicoides* biting midges for the presence of pathogens is a cost-effective method of identifying potential vectors which can then be targeted for more detailed laboratory studies to prove their vector competence. Knowledge of the vector is a key factor to assess the epidemiology of these diseases for better prevention and control measures in Thailand.
